# Biotransformation of Silymarin Flavonolignans by Human Fecal Microbiota

**DOI:** 10.3390/metabo10010029

**Published:** 2020-01-09

**Authors:** Kateřina Valentová, Jaroslav Havlík, Pavel Kosina, Barbora Papoušková, José Diógenes Jaimes, Kristýna Káňová, Lucie Petrásková, Jitka Ulrichová, Vladimír Křen

**Affiliations:** 1 Laboratory of Biotransformation, Institute of Microbiology of the Czech Academy of Sciences, Vídeňská 1083, 14220 Prague, Czech Republic; astriik@gmail.com (K.K.); petraskova@biomed.cas.cz (L.P.); kren@biomed.cas.cz (V.K.); 2 Department of Food Science, Czech University of Life Sciences Prague, Kamýcká 129, 16500 Prague, Czech Republic; havlik@af.czu.cz (J.H.); jose.d.jaimes@gmail.com (J.D.J.); 3 Department of Medical Chemistry and Biochemistry, Faculty of Medicine and Dentistry, Palacký University, Hněvotínská 3, 77515 Olomouc, Czech Republic; pavel.kosina@upol.cz (P.K.); jitka.ulrichova@upol.cz (J.U.); 4 Regional Centre of Advanced Technologies and Materials, Department of Analytical Chemistry, Faculty of Science, Palacký University, 17. listopadu 12, 77146 Olomouc, Czech Republic; barbora.papouskova@upol.cz; 5 Department of Biochemistry and Microbiology, University of Chemistry and Technology Prague, Technická 5, CZ 16628 Prague, Czech Republic

**Keywords:** silymarin, flavonolignans, biotransformation, metabolites, gut microbiota, UHPLC–MS, inter-individual differences

## Abstract

Flavonolignans occur typically in *Silybum marianum* (milk thistle) fruit extract, silymarin, which contains silybin, isosilybin, silychristin, silydianin, and their 2,3-dehydroderivatives, together with other minor flavonoids and a polymeric phenolic fraction. Biotransformation of individual silymarin components by human microbiota was studied ex vivo, using batch incubations inoculated by fecal slurry. Samples at selected time points were analyzed by ultrahigh-performance liquid chromatography equipped with mass spectrometry. The initial experiment using a concentration of 200 mg/L showed that flavonolignans are resistant to the metabolic action of intestinal microbiota. At the lower concentration of 10 mg/L, biotransformation of flavonolignans was much slower than that of taxifolin, which was completely degraded after 16 h. While silybin, isosilybin, and 2,3-dehydrosilybin underwent mostly demethylation, silychristin was predominantly reduced. Silydianin, 2,3-dehydrosilychristin and 2,3-dehydrosilydianin were reduced, as well, and decarbonylation and cysteine conjugation proceeded. No low-molecular-weight phenolic metabolites were detected for any of the compounds tested. Strong inter-individual differences in the biotransformation profile were observed among the four fecal-material donors. In conclusion, the flavonolignans, especially at higher (pharmacological) doses, are relatively resistant to biotransformation by gut microbiota, which, however, depends strongly on the individual structures of these isomeric compounds, but also on the stool donor.

## 1. Introduction

**Figure 1:**
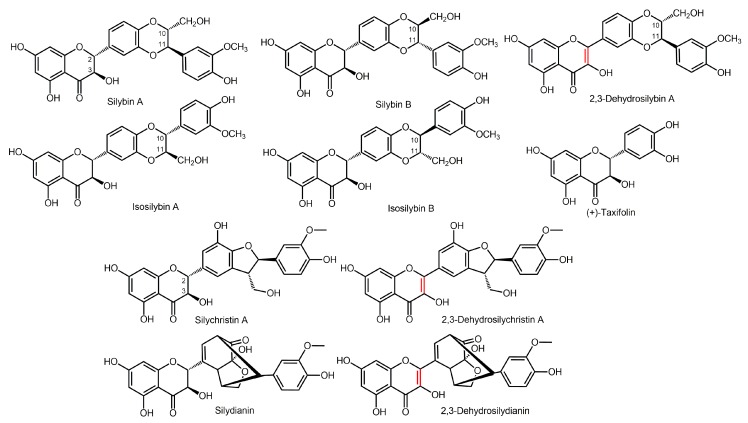
Structures of (selected) silymarin components.

Flavonolignans are plant secondary metabolites belonging to the group of polyphenols formed by the coupling of a flavonoid with a phenylpropanoid (lignan); they occur typically in *Silybum marianum* (L.) Gaertn (milk thistle, Asteraceae) fruits (cypselae). The crude extract denoted as silymarin is used in a plethora of nutraceutical preparations and drugs, mainly for its hepatoprotective action [1]; nonetheless, it also exerts a number of other pharmacological activities, such as cardioprotective [2], hypocholesterolemic [3], and antidiabetic [4,5] activities. Silymarin contains over ten structurally related flavonolignans, which are all biogenetic congeners [6]. Major constituents of silymarin are as follows: flavonolignans silybin A and B; isosilybin A and B; silychristin A; silydianin; the flavanol (+)-taxifolin [7]; and ca 30% of a polyphenolic fraction. Minor silymarin flavonolignans include 2,3-dehydrosilybin [8,9], 2,3-dehydrosilychristin, and 2,3-dehydrosilydianin [10] (Figure 1). Silybin is the most studied compound of silymarin, since it is assumed to be its active substance. Studies have shown its great potential in the treatment of Alzheimer’s disease [11], as well as in cancer therapy [12,13,14]. Although manufacturers often claim their preparations to contain a “standardized” extract, the composition of silymarin and silymarin-containing food supplements and drugs differ profoundly, even batch to batch for the same preparation [6].

To exert their biological activity in a human or animal organism, the molecule of interest needs to be bioavailable. Polyphenols in general suffer from low bioavailability and excessive metabolism by mammalian enzymes mostly in intestinal and hepatic cells [15]. Even though little is known to date about the metabolic fate of flavonolignans, they were found to be metabolized by human liver enzymes [16,17,18,19]. Silybin was shown to undergo both phase I oxidative metabolism and phase II conjugation in vitro. The methoxy group at silybin C-19 was *O*-demethylated by human cytochrome P450 (CYP) 2C8 [20]. Silybin may also be glucuronidated at C-7-OH and C-20-OH groups by human UDP-glucuronosyltransferases (UGTs) 1A and 2B [21,22], and sulfated metabolites have been identified in human plasma [16]. By the use of perfused rat liver, the formation of sulfated and glucuronidated conjugates of silychristin and silydianin was observed, as well [17]. In addition, our results from the study on human hepatocytes and microsomes indicates that the flavonolignans are primarily metabolized by phase II conjugation enzymes [18,19].

However, a large proportion of (poly)phenolic compounds is known not to be resorbed in the upper part of the gastrointestinal tract; they are transported to the lower intestine and metabolized by gut microbiota [15,23]. As far as we know, this aspect has been studied to a very limited extent with the flavonolignans. The only study available to date focused on the biotransformation of silymarin complex by an intestinal bacterial strain *Eubacterium limosum* with demethylation activity and described the formation of demethylated flavonolignan derivatives [24]. Although it is not focused on biotransformation, a recent mice study investigating silymarin and silybin as potential microbiota-based therapy of Alzheimer’s disease suggests that their administration could alleviate memory impairment in pathological mice, which was accompanied by their regulative effect on the gut microbiota composition, specifically on an enrichment of the phyla *Verrucomicrobia* [25]. Just like with other polyphenols, this points to a two-way relationship between the microbiota and silymarin components; however, detailed information on the biotransformation of silymarin components by human gut microbiota is still missing. The aim of the present study was to investigate metabolic biotransformation of isolated silymarin flavonolignans by human fecal slurry from healthy donors [26].

## 2. Results and Discussion

### 2.1. Pilot Study

Silybin, silychristin, silydianin, and 2,3-dehydrosilybin were treated under simulated gastric and subsequently small intestinal conditions, using a standardized static in vitro digestion method [27], in order to evaluate the possible effects of pH alteration and the presence of ions or nonspecific pancreatic esterases. Nevertheless, no modifications, such as partial degradation, polymerization, or complexation, were observed during the oral and/or intestinal phase.

**Figure 2:**
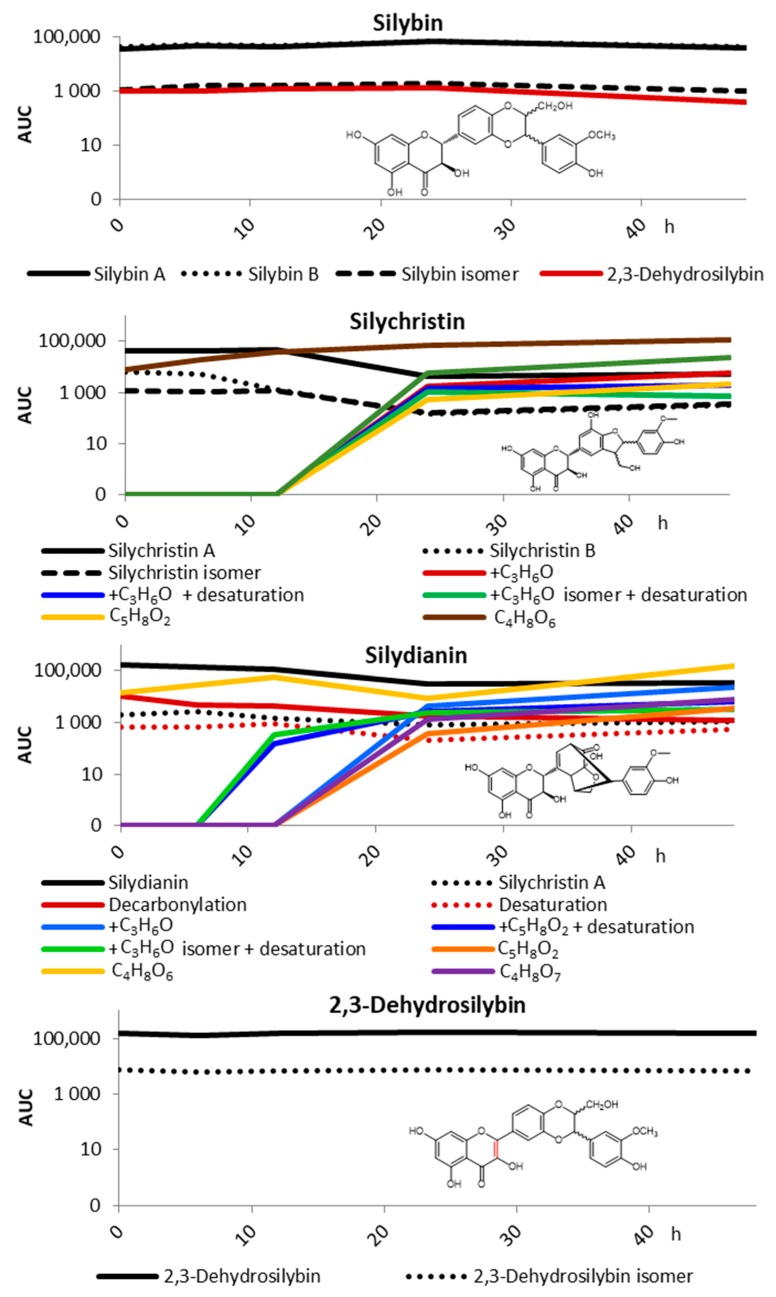
Pilot biotransformation of selected silymarin components (200 mg/L) by human fecal microbiota after pre-incubation in simulated gastric and low intestinal conditions. The results are shown as the area under the curve (AUC) of the respective peak in LC/MS chromatograms (logarithmic scale) as a function of time of incubation; minor metabolites with AUC < 1% were excluded for better clarity.

The digestate containing 200 mg/L of the respective flavonolignan was incubated under anaerobic conditions in a fecal-batch incubation model for up to 48 h. Such a high dose was used because silymarin is often consumed in food supplement capsules packed with up to 500 mg of extract. Considering that this amount reaches the caecum in a bolus and it is diluted with ca 200 mL of the cecal content [28], it may reach the concentration of the extract up to 2.5 g/L. The dose used in this pilot experiment was one order of magnitude lower, to reflect the content of the individual components in silymarin (0.5%–30%), but it still exceeds the concentrations used by other studies [29], and it is close to minimal inhibitory concentrations toward some microbial taxa [30,31,32]. Concentrations found in the incubation medium after the pre-analytical stage, including precipitation of proteins with acetonitrile and centrifugation, were much lower and different for the individual compounds (Table 1), potentially due to sequestration to macromolecules or solubility issues. This is in agreement with previously published data on the binding of polyphenols, including silybin to macromolecules or cell membranes [33,34,35,36]. The data also revealed that, at the dose used, the concentration of both silybin and 2,3-dehydrosilybin in the medium remained unchanged during the experiment. On the other hand, silychristin and silydianin were intensively metabolized, and after 48 h, their concentration dropped to 13% and 21% of their initial value, respectively (Table 1 and Figure 2).

The spectrum of the metabolites found was quite broad, and as standards were not available for all of them, the results are presented as the area under the curve (AUC) of the respective peak in LC/MS chromatograms (Figure 2). The analysis was primarily focused on the metabolites produced by usual metabolic pathways and having molecular masses close to that of the parent compounds, using a commercial software (Metabolynx V4.1 software, Waters), as well as detailed manual study based on UV chromatograms obtained from a PDA detector. We found intensive adducts of silychristin and silydianin with *m*/*z* increment corresponding to the group/exact mass of C_3_H_6_O; in the case of 2,3-dehydrosilybin, the addition of C_3_H_4_O was found as a minor biotransformation product. Both the parent compounds and adducts were further desaturated, acetylated, demethylated and/or reduced. However, the (estimated) amount of these metabolites did not correspond to the decrease in the concentration of the parent compounds, indicating alternative pathways. Therefore, we looked at potential lower molecular degradation products similar to those described for various flavonoids, such as quercetin, kaempferol, luteolin, or catechin [37,38,39,40]. Indeed, two low-molecular products with *m*/*z* 151 and 167 were identified as a result of silychristin and silydianin biotransformation (Figure 2). However, these products corresponded to C_4_H_8_O_6_ and C_4_H_8_O_7_, respectively, instead of the expected simple phenolics, such as hydroxyphenylacetic and dihydroxyphenylacetic acids. Their structures could not be inferred from available data.

### 2.2. Fecal Fermentation of Silymarin Flavonolignans Ex Vivo

Based on the results of the pilot study, and in line with recently published findings [41], the pre-incubation was skipped for the following study using fecal samples from four healthy individuals. The medium used here was a standard medium employed previously in similar fermentation studies [26]. A complex panel of silymarin constituents, i.e., taxifolin, silybin, isosilybin, silychristin, silydianin, 2,3-dehydrosilybin, 2,3-dehydrosilychristin, and 2,3-dehydrosilydianin, was subjected to biotransformation at a lower final concentration of 10 mg/L, which corresponds rather to the typical dietary intake of these phenolics [41].

**Figure A1:**
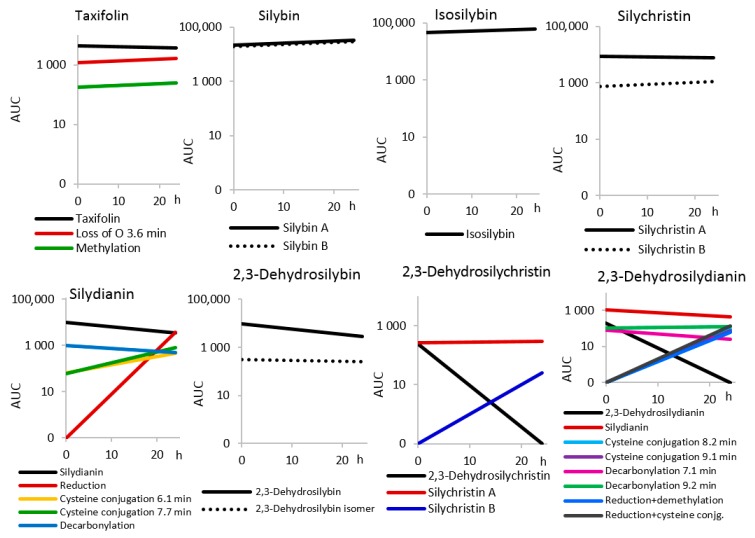
Stability of the compounds tested in the incubation medium without fecal microbiota.

In this setup, we first tested the stability of all compounds, which were incubated in the medium without the fecal slurry under the same (anaerobic) conditions. While silybin, isosilybin, and silychristin remained stable during 24 h of incubation, a slight decrease after 24 h was observed for taxifolin (14%). On the other hand, substantial degradation occurred with 2,3-dehydrosilybin (71%), silydianin (64%), and especially 2,3-dehydrosilychristin and 2,3-dehydrosilydianin, which completely disappeared from the incubation mixture. Moreover, significant degradation of silydianin, 2,3-dehydrosilydianin, and partially also of 2,3-dehydrosilychristin and taxifolin was noted also at t = 0 h (Figure A1). This may be due to inherent instability of the compounds during the pre-analytical stage of the sample preparation. The purity of the compounds in solid state was retested after the experiment, and it was identical to the values obtained prior to the experiment (see Section 3.1).

The fermentation of the compounds at such low concentrations and in absence of glucose brought about significant differences compared to the pilot study, as no adducts with C_3_H_6_O or C_3_H_4_O were formed. Moreover, focusing on the unidentified metabolites C_4_H_8_O_6_ and C_4_H_8_O_7_, although they were detected in the fermentation media of all samples, we observed that their content was relatively high in the beginning of the fermentation and decreased over time (data not shown). Therefore, we conclude that these compounds were probably not metabolites of the flavonolignans.

**Figure 3:**
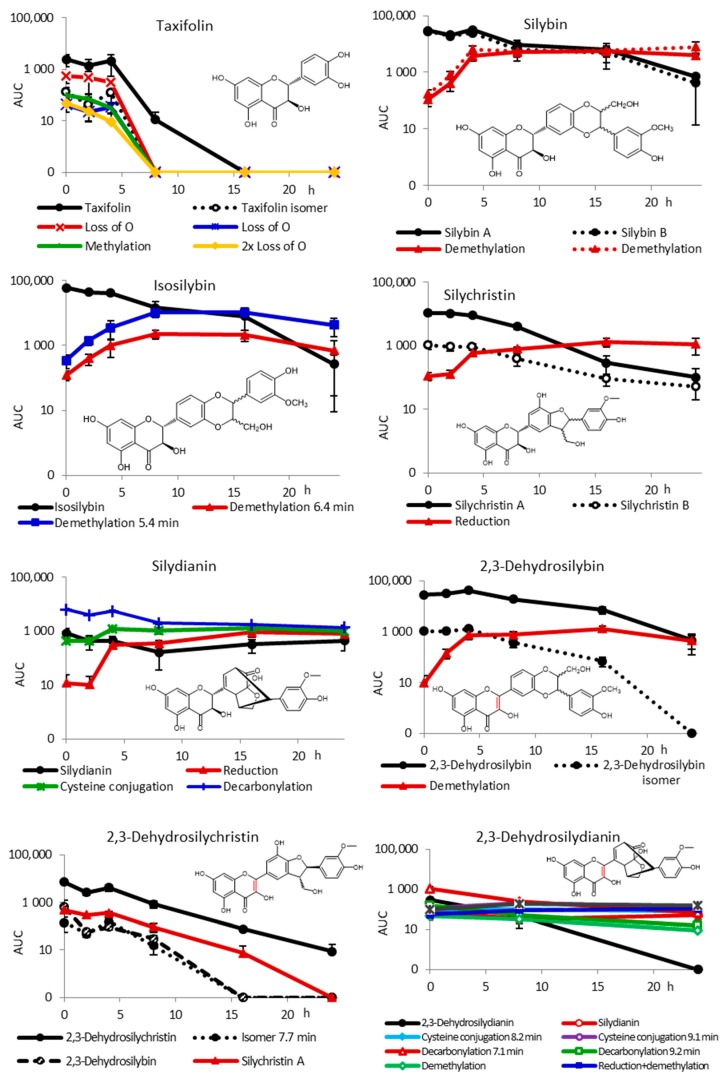
Biotransformation of silymarin components (10 mg/L) by human fecal microbiota from four healthy donors without pre-incubation under simulated gastric and subsequently small-intestinal conditions. The results are shown as the area under the curve (AUC) of the respective peak in LC/MS chromatograms (logarithmic scale) as a function of time of incubation (mean ± SEM, *n* = 4); minor metabolites with AUC < 1% were excluded for better clarity.

Further, the results (Figure 3) show an important difference in the metabolic fate of flavanonols represented by taxifolin compared with flavonolignans. Taxifolin was substantially (99.5%) degraded already after 8 h of incubation, while the metabolism of flavonolignans was much slower. Major taxifolin metabolites detected involved its reduced (loss of O), methylated, and 2× loss of O products (Table A1), which all disappeared after 8 h of incubation, as well. No phenolic compounds of lower molecular weight were detected. The degradation products might have been catabolized by the bacteria, sequestered on the matrix or adsorbed on cell walls.

Silybin was found to be primarily demethylated (probably at C-19), both diastereomers to the same extent and at the same rate. The final amount of silybin A and B was 2% and 1.5% of the initial concentration, respectively. The biotransformation pattern of isosilybin was similar to that of silybin, with the exception that the diastereomers of the parent compound were not always sufficiently separated by using the method used. Less than 1% of the initial amount of the parent compound was present in the medium after 24 h of incubation. Two demethylation products with very similar retention times (RT) of 6.44 and 6.54 min, probably those of the two diastereomers, both at C-19 (Table A1), were formed as major metabolites.

In the case of silychristin, in line with the pilot study, the biotransformation was faster with less than 3% of the parent compound at 16 h already. Here, the major metabolite was not a demethylation product, but a reduced derivative eluting at 5.30 min different from 2,3-dehydrosilychristin. In the case of silydianin, the analysis of the data was complicated due to the compound decomposition, which occurred probably during sample manipulation. The content of the parent compound in the medium was only 9% of all silydianin derivatives at time t = 0 h, and it further decreased during incubation. The major, mostly spontaneously formed product was its decarbonylated derivative, which accounted for 68% at t = 0 h and was further degraded. A hydrogenated derivative, presumably identical to that one produced previously by human hepatocytes [18], was the only metabolite whose concentration gradually increased during incubation and reached 19% at t = 24 h. Moreover, another derivative corresponding to a cysteine conjugation product was detected for the first time whose content gradually increased up to 35% after 24 h.

The biotransformation pattern of 2,3-dehydrosilybin was very similar to that of silybin, where the major metabolite was a demethylated derivative (individual enantiomers were not separated). Besides 2,3-dehydrosilybin, minor amounts of another dehydroflavonolignan were present at t = 0 h (3.6%, RT 9.81 min, Table A1), which was degraded during fermentation. In the case of 2,3-dehydrosilychristin, no clear metabolite was found, as all compounds were present already at t = 0 h and disappeared gradually. The most complex mixture of products was found for 2,3-dehydrosilydianin. Besides the parent compound, various cysteine conjugation (8.2 and 9.1 min), decarbonylation (7.1 and 9.2 min), demethylation, reduction + demethylation, and reduction + cysteine conjugation products (Table A1) were found. However, all these were formed also during the stability test, and the AUC of all these compounds was only 4% of that found for silybin or isosilybin. The results for 2,3-dehydrosilydianin are therefore inconclusive.

### 2.3. Inter-Individual Variability

**Figure A2:**
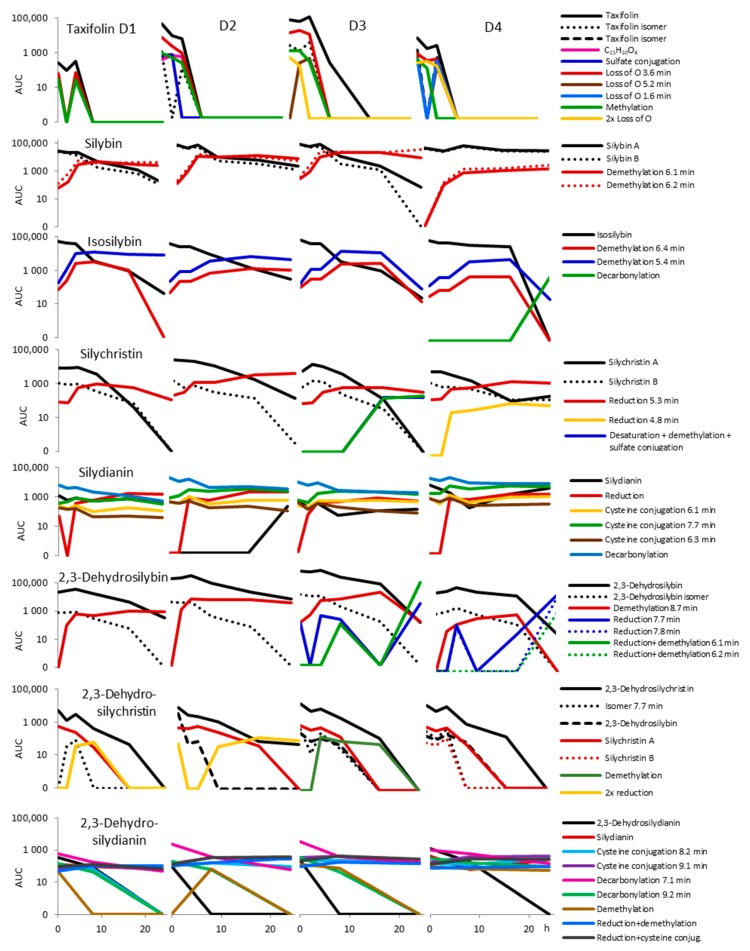
Biotransformation of silymarin components by human fecal microbiota of individual donors D1, D2, D3, and D4 (*cf*. Figure 3).

Although the volunteers providing stool samples for this study were quite homogenous in age and BMI, the ability of their gut microbiota to metabolize the flavonolignans differed substantially, resulting in high standard errors of mean (SEM, Figure 3) and large inter-individual variability (Figure A2). Not only the degradation rate differed, but some metabolites were observed only in some volunteers. For instance, isosilybin was decarbonylated only with fecal slurry from donor D4, silychristin was desaturated + demethylated and eventually sulfated in the case of donor D3, and an additional reduction product eluting at 4.8 min was found for donor D4. For 2,3-dehydrosilybin additional reduction and reduction + demethylation products were observed for donors D3 and D4 (Figure A2). This is in accordance with the previous studies using other natural compounds, such as stilbenoids [42].

## 3. Materials and Methods

### 3.1. Flavonolignans and Other Chemicals

Natural silybin (containing 55% of silybin A, 44% of silybin B, 0.6% of isosilybin A, and 0.4% of isosilybin B) was isolated from silymarin by its quick suspending in methanol and filtration yielding undissolved silybin. Isosilybin containing 22.7% of isosilybin A, 72.7% of isosilybin B, 1.2% of silybin A, and 1.7% of silybin B was isolated by using enzymatic kinetic resolution of silymarin mixture [43]. Silychristin (containing 83.4% of silychristin A, 10.2% of silychristin B, 3.1% of taxifolin, 1.1% of isosilychristin, and 1.4% of 2,3-dehydrosilychristin) and silydianin (98.8% containing 1.2% of silychristin for the pilot study, 100% for the main experiment) were isolated from silymarin, using Sephadex LH-20 chromatography [7]. Then, 2,3-Dehydrosilybin (37% of 2,3-dehydrosilybin A, 62% of 2,3-dehydrosilybin B), 2,3-dehydrosilychristin (96.6%, containing 3.4% of silychristin), and 2,3-dehydrosilydianin (84%) were prepared as described previously [10]. Taxifolin (95.5% containing 4.5% of quercetin) was purchased from Amagro (Prague, Czech Republic).

The purity of the flavonolignans and taxifolin was measured by using the HPLC gradient method [44] on a Shimadzu Prominence LC analytical system consisting of LC-20AD binary HPLC pump, CTO-10AS column oven, SIL-20ACHT cooling auto sampler, CBM-20A system controller, and SPD-20MA diode array detector (Shimadzu, Kyoto, Japan): a Chromolith RP-18e (100 × 3 mm) column equipped with a Chromolith RP-18e guard cartridge (5 × 4.6 mm), mobile phase A = 5% acetonitrile in water, 0.1% HCOOH; mobile phase B = 80% acetonitrile in water, 0.1% HCOOH; gradient: 0–5 min 0%–25% B, 5–8 min 25%–60% B, 8–10 min 60% B, 10–11 min 60%–0% B, and 12 min stop. Flow rate was 1.2 mL/min, and temperature was 25 °C. For 2,3-dehydroflavonoligans, an LC–MS method on a Chromolith RP-18e (100 × 3 mm) column and Chromolith RP-18e (5 × 4.6 mm) precolumn (Merck, Darmstadt, Germany) was used, mobile phase: A = 5% acetonitrile, 0.1% HCOOH; B = 80% acetonitrile, 0.1% HCOOH; gradient: 0 min 20% B, 5 min 90% B, 6 min 90% B, 8–10 min 20% B; flow rate 0.4 mL/min, 25 °C, MS detection. The MS parameters were as follows: negative mode; ESI interface voltage, 4.5 kV; detector voltage, 1.15 kV; nebulizing gas flow, 1.5 mL/min; drying gas flow, 15 mL/min; heat block temperature, 200 °C; DL temperature, 250 °C; SCAN mode 450–650 *m*/*z*; spectra were extracted in the 479.0–479.1 *m*/*z* range ([M–H]^−^ ions of dehydrocompounds). Software LabSolutions ver. 5.75 SP2 (Shimadzu, Kyoto, Japan). The NMR and MS spectra of compounds used were identical to the authentic standards available in the Laboratory of Biotransformation, Institute of Microbiology of the CAS, Prague.

Stock solutions for the fermentation experiments (Section 3.4) were prepared at a concentration of 10 mg/mL in DMSO (dimethylsulfoxide; Sigma-Aldrich, Prague, Czech Republic) and kept at 4 °C. Analytical standard stock solutions for LC/MS were prepared in methanol (1 mg/mL) and stored at −80 °C. Other chemicals were obtained from Merck (Darmstadt, Germany).

### 3.2. Fecal Samples and Ethics Statement

The study was performed in accordance with the Declaration of Helsinki, and the protocol was approved by the Ethics Committee of the Czech University of Life Sciences in Prague, Czech Republic (ZEK/22/09/2017). All subjects gave their informed consent for inclusion in the study. Human fecal samples were collected at the Czech University of Life Sciences, from a total of 1 healthy male volunteer donor for the pilot study and 4 healthy male volunteer donors for the fecal fermentation. Donors ranged from 26 to 41 years, their BMI was within the normal range (18.5–24.9), and they had no history of gastrointestinal disease and no antibiotic treatment for at least 3 months prior to the experiment. The donors followed an omnivorous diet in their daily life. Feces were collected into 1 L plastic containers, tied in plastic bags with GENbag anaer (Biomérieux, Lyon, France), kept at 37 °C for 2 h maximum, and then homogenized for 30 s with a sodium phosphate buffer (1/15 M, pH 7, previously boiled with cotton cups and cooled to approximately 37 °C, while purged with N_2_ free of oxygen) in a stomacher bag. The obtained 32% fecal slurry was filtrated through nylon mesh.

### 3.3. Pilot Study

Silymarin flavonolignans silybin, silychristin, silydianin, and 2,3-dehydrosilybin (20 mg) were mixed with 5 mL of the gastric phase solution, i.e., a mixture of 1.25 mL of simulated gastric fluid (SGF, containing 6.9 mM KCl, 0.9 mM KH_2_PO_4_, 25 mM NaHCO_3_, 47.2 mM NaCl, 0.1 mM MgCl_2_, and 0.5 mM (NH_4_)_2_CO_3_), 1.25 mL of pepsin solution (0.0008 g/1.25 mL), and 2.5 mL of 0.15 mM CaCl_2_; pH 3. Gastric phase was simulated via 2 h of shaking (200 rpm) in 50 mL Falcon tubes at 37 °C. Then the resulting mixture was stirred with a total 5 mL of the ileal phase, i.e., a mixture of 0.83 mL of simulated ileal fluid (SIF, containing 6.8 mM KCl, 0.8 mM KH_2_PO_4_, 85 mM NaHCO_3_, 38.4 mM NaCl, and 0.33 mM MgCl_2_), pancreatin solution (0.0008 g/0.83 mL), bile solution (0.0067 g/0.83 mL), and 2.5 mL of 0.6 mM CaCl_2_·H_2_O. The pH of the final mixture was set to 7, and the ileal phase was simulated via 2 h shaking (200 rpm) in 50 mL Falcon tubes at 37 °C. Aliquots (1 mL) after the ileal digestion phase of were mixed in 10 mL vials with 9 mL of sterile brain heart infusion (BHI, 37 g/L) medium with hemin (5 mg/L) and yeast extract (5 g/L), pH 7.4, previously reduced by bubbling with CO_2_ and kept in anaerobic conditions (5% CO_2_, 10% H_2_, and 85% N_2_) for 48 h prior the experiment, inoculated with the donor stool (1%), and cultured for 48 h in duplicates in a shaking water bath at 37 °C. Samples containing a final concentration of 0.2 mg/mL of the respective flavonolignan or buffer as the negative control were taken at 0, 6, 12, 24, and 48 h, frozen, and kept at −80 °C prior to processing.

### 3.4. Fecal Fermentation of Silymarin Flavonolignans Ex Vivo

The fermentations were carried out by using 96-well plastic plates (2.2 mL) and fecal slurries from four different healthy volunteers. The fermentation medium was prepared as previously described [26] by mixing 225 mL of distilled water, 1.12 g of tryptone, 56.25 µL of micromineral solution (0.9 mM CaCl_2_; 0.5 mM MnCl_2_; 0.042 mM CoCl_2_; and 3 mM FeCl_3_), and 112.5 mL of macromineral solution (40 mM Na_2_HPO_4_; 45.6 mM KH_2_PO_4_; and 2.4 mM MgSO_4_); 112.5 mL of carbonate buffer (41.7 mM NH_4_HCO_3_ and 41.7 mM NaHCO_3_), and 562.5 µL of 0.1% resazurin solution was boiled with cotton stoppers and cooled to approximately 37 °C, while purged with oxygen-free N_2_. Glucose was absent in the medium to enforce utilization of the phenolics as a sole carbon source. The fermentation medium (1.68 mL) was transferred into each well of the deep-well plate. Additionally, 80 μL of the reducing solution (0.8 mL 1M NaOH and 125 mg Na_2_S in 20 mL of H_2_O). Then the tested compounds solution (40 µL), initially dissolved in DMSO, were added so that the final concentration in total 2 mL volume was 105 mg/L. Fermentation was started with the addition of 2 mL of the fecal slurry. The well plates were then covered and placed inside a vacuum sealer bag, along with an Anaerogen sachet (Oxoid CZ, Brno, Czech Republic) and an anaerobic indicator. The bags were sealed and placed inside a 37 °C incubator on top of a shaker (100 strokes/min). Time point 0 h was placed directly in a −80 °C freezer. At 0, 2, 4, 8, 16, and 24 h, the corresponding well plate was removed from the incubator and placed inside a −80 °C freezer.

### 3.5. Sample Preparation

Samples stored at −80 °C were thawed and mixed with acetonitrile (cooled at −20 °C) for 1 h, at −20 °C, to precipitate the proteins. After centrifugation (12,000× g, 5 min), the sediment was washed with cooled acetonitrile and centrifuged again, and the supernatants were combined for HPLC/MS analysis. Before the analysis, the samples were stored at −80 °C.

### 3.6. Analysis of the Metabolites

Ultrahigh-performance liquid chromatography with mass spectrometry (UHPLC-MS) was performed using Waters ACQUITY I-Class UPLC system (Waters, Milford, MA, USA) consisting of a binary solvent manager, sample manager, column manager and PDA detector. The chromatographic separation was achieved on Kinetex Biphenyl analytical column (100 mm × 2.1 mm i.d., 1.7 µm; Phenomenex, Torrance, CA, USA). The injection volume was 2 µL. Solvent A was composed of 5 mM ammonium acetate, pH 3, and solvent B was methanol. The linear gradient profile was as follows: 0–8 min 30%–60% B, 8–13 min 60%–90% B, 13–14 min 90% B, and 14–15 min 90%–30% B. The mobile phase flow rate was 0.35 mL/min, the temperature of the autosampler was maintained at 4 °C and the column oven was set to 40 °C.

**Figure A3:**
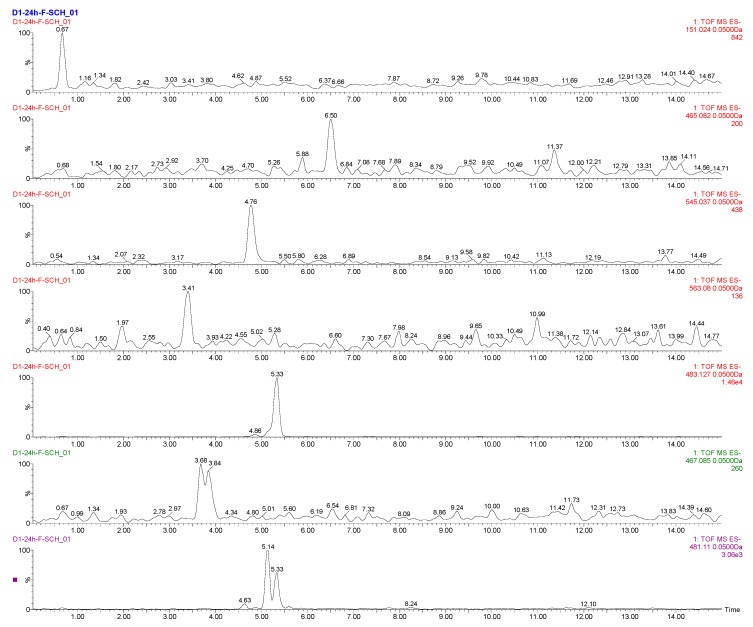
An example of UHPLC–MS chromatograms for the fermentation of silychristin by fecal microbiota from donor D4 for 24 h.

A Waters Synapt G2-S Mass Spectrometer (Waters, Manchester, UK) was connected to the UPLC system via an electrospray ionization (ESI) interface. The ESI source operated in negative mode with the capillary voltage at 2.25 kV and the sampling cone at 35 V. The source temperature and the desolvation temperature were set at 120 and 300 °C, respectively. The cone and desolvation gas flows were 25 and 600 L/h, respectively. Data were acquired from 100 to 1200 Da, with a 0.2 s scan time. The mass spectrometer was calibrated across the mass range 100–1200 Da, using a solution of sodium formate in acetonitrile. Data were automatically centroided and mass corrected during acquisition, using a leucine–enkephalin external reference (20 µg/L in a mixture of water/acetonitrile/formic acid (100:100:0.2), flow rate of 10 µL/min). Data acquisition was accomplished by using two interleaved scan functions (MS^E^ experiments), which enabled simultaneous acquisition of both low-collision-energy (CE) and high-collision-energy mass spectra from a single experiment. The low trap CE was set to 4 V, and the low transfer CE was set to 2 V for Function 1. For Function 2 (high CE), the trap CE was set to 4 V, and the transfer lenses were ramped CE in the range of 15–30 V. Post-acquisition processing of the data was performed by using the Metabolynx V4.1 software (Waters). Typical chromatograms are given in Figure A3.

## 4. Conclusions

To the best of our knowledge, this is the first study evaluating the biotransformation of individual silymarin constituents by human fecal microbiota, with detailed characterization of individual studied flavonolignans purity and diastereomeric/enantiomeric ratio. Our results show that, in contrast to simple flavonoids such as flavan-3-ols, flavonols, flavones, and flavanones or phenolic acids [26,38,39,45,46,47,48,49], the flavonolignans, especially at higher doses, achievable using silymarin containing food supplements and drugs, are fairly resistant to the metabolic action of intestinal microbiota. The metabolic profiles, however, not only strongly depend on the individual structures of these isomeric compounds, but also on the strong inter-individual differences in microbial composition. Based on this fact, our further investigation will be carried out with a larger group of individual volunteers.

## Figures and Tables

**Table 1:** The concentration (µM) of the parent flavonolignans during the fecal biotransformation.

Compound	0	6	12	24	48 h
Silybin A	7.0 ± 1.2	7.6 ± 0.8	7.8 ± 1.2	10.4 ± 0.5	8.5 ± 1.8
Silybin B	5.8 ± 1.1	6.7 ± 0.7	6.5 ± 1.1	9.5 ± 0.8	6.7 ± 2.0
Silychristin A	29.0 ± 0.5	29.6 ± 0.4	33.5 ± 1.3	5.1 ± 1.2	3.5 ± 0.2
Silychristin B	4.8 ± 0.1	3.8 ± 0.1	1.2 ± 0.1	0.4 ± 0.0	0.5 ± 0.1
Silydianin	69.9 ± 0.9	70.1 ± 8.0	55.8 ± 5.4	19.7 ± 3.6	13.1 ± 1.0
2,3-Dehydrosilybin (A + B)	57.4 ± 3.8	47.8 ± 2.3	54.2 ± 1.0	60.6 ± 5.4	57.5 ± 1.1

Data are presented as mean ± standard error, *n* = 3.

## Appendix A

**Table A1:** LC/MS parameters of flavonolignans and their metabolites produced by human fecal microbiota.

Compound	Elemental Composition	*m*/*z*	t_R_ (min)	Δppm	MS^E^
**Taxifolin**					
Taxifolin	C_15_H_12_O_7_	303.0490	2.5	−4.9	175.0391; 125.0224
Taxifolin isomer	C_15_H_12_O_7_	303.0486	2.8	−6.3	125.0221
C_15_H_10_O_4_	C_15_H_10_O_4_	253.0488	10.3	−5.1	xxx
Sulfate conjugation	C_15_H_12_O_10_S	383.0034	1.5	−7.3	285.0424
Loss of O	C_15_H_12_O_6_	287.0540	3.6	−5.6	177.0523; 125.0225
Loss of O	C_15_H_12_O_6_	287.0538	5.2	−6.3	xxx
Loss of O	C_15_H_12_O_6_	287.0541	1.6	−5.2	xxx
Methylation	C_16_H_14_O_7_	317.0650	4.2	−3.5	125.0263
2x Loss of O	C_15_H_12_O_5_	271.0609	6.8	1.1	xxx
**Silybin**					
Silybin A	C_25_H_22_O_10_	481.1118	7.7	−3.5	301.0304; 125.0217
Silybin B	C_25_H_22_O_10_	481.1123	7.8	−2.5	301.0349; 125.0210
Isosilybin	C_25_H_22_O_10_	481.1165	8.2	6.2	xxx
Demethylation	C_24_H_20_O_10_	467.0946	6.1	−6.9	301.0317; 125.0212
Demethylation	C_24_H_20_O_10_	467.0964	6.2	−3.0	301.0322; 125.0217
**Isosilybin**					
Isosilybin	C_25_H_22_O_10_	481.1133	8.3	−0.4	453.1169; 125.0224
Demethylation	C_24_H_20_O_10_	467.0954	6.4	−5.1	125.0221
Demethylation	C_24_H_20_O_10_	467.0969	6.5	−1.9	285.0389; 125.0227
Decarbonylation	C_24_H_21_O_9_	453.1164	6.3	−4.9	181.0141; 125.0203
**Silychristin**					
Silychristin A	C_25_H_22_O_10_	481.1129	5.1	−1.2	325.0701; 125.0232
Silychristin B	C_25_H_22_O_10_	481.1124	5.3	−2.3	325.0687
Reduction	C_25_H_24_O_10_	483.1268	5.3	−4.8	357.0912; 125.0242
Reduction	C_25_H_24_O_10_	483.1346	4.8	11.4	xxx
Desaturation + demethylation + sulfate conjugation	C_24_H_18_O_13_S	545.0369	4.8	−3.9	xxx
Desaturation + demethylation	C_24_H_18_O_10_	465.0819	6.5	−0.6	xxx
+ C_3_H_6_O	C_28_H_28_O_11_	539.1550	5.9	−0.6	183.0648; 237.0384
+ C_3_H_6_O + desaturation	C_28_H_26_O_11_	537.1390	7.0	−1.3	235.0247
+ C_3_H_6_O isomer + desaturation	C_28_H_26_O_11_	537.1394	7.3	−0.6	183.0655; 235.0224
+ C_5_H_8_O_2_	C_30_H_30_O_12_	581.1659	6.2	−0.2	553.1713; 279.0490
C_4_H_8_O_6_	C_4_H_8_O_6_	151.0239	0.7	−2.6	xxx
C_4_H_8_O_7_	C_4_H_8_O_7_	167.0200	0.6	4.8	xxx
**Silydianin**					
Silydianin	C_25_H_22_O_10_	481.1135	6.6	0.0	178.9972; 151.0023
Reduction	C_25_H_24_O_10_	483.1280	4.7	−2.3	125.0236
Cysteine conjugation	C_28_H_27_NO_11_S	584.1212	6.1	−2.6	326.0632; 178.9949
Cysteine conjugation	C_28_H_27_NO_11_S	584.1229	7.7	0.3	125.0215; 326.0641
Cysteine conjugation	C_28_H_27_NO_11_S	584.1216	6.3	−1.9	326.0647; 125.0211
Cysteine conjugation	C_28_H_27_NO_11_S	584.1207	6.0	−3.4	326.0670
Decarbonylation	C_24_H_22_O_9_	453.1169	6.3	−3.8	125.0215; 182.0191
Desaturation	C_25_H_20_O_10_	479.0982	7.2	0.8	299.0183
+C_3_H_6_O	C_28_H_28_O_11_	539.1563	6.9	−0.6	209.0441; 237.0386
+C_3_H_6_O + desaturation	C_28_H_26_O_11_	537.1393	7.7	−0.7	235.0241
+C_3_H_6_O isomer + desaturation	C_28_H_26_O_11_	537.1396	8.2	−0.2	235.0220; 207.0274
+ C_5_H_8_O_2_	C_30_H_30_O_12_	581.1656	7.2	−0.5	xxx
C_4_H_8_O_6_	C_4_H_8_O_6_	151.0252	0.8	6.0	135.0308
C_4_H_8_O_7_	C_4_H_8_O_7_	167.0205	0.7	7.8	xxx
**2,3-Dehydrosilybin**					
2,3-Dehydrosilybin	C_25_H_20_O_10_	479.0985	10.2	1.5	299.0185; 271.0237
2,3-Dehydrosilybin isomer	C_25_H_20_O_10_	479.0971	10.0	−1.5	299.0186; 271.0241
Demethylation	C_24_H_18_O_10_	465.0825	8.7	0.6	299.0188; 271.0238
Silybin A	C_25_H_22_O_10_	481.1115	8.0	−4.2	301.0331; 125.0248
Silybin B	C_25_H_22_O_10_	481.1134	8.1	−0.2	301.0341
Reduction + demethylation	C_24_H_20_O_10_	467.0970	6.1	−1.7	125.0215; 301.0320
Reduction + demethylation	C_24_H_20_O_11_	467.0963	6.2	−3.2	125.0213; 301.0315
**2,3-Dehydrosilychristin**					
2,3-Dehydrosilychristin	C_25_H_20_O_10_	479.0964	7.4	−2.9	449.0852; 151.0018
Dehydrosilychristin isomer	C_25_H_20_O_10_	479.0958	7.7	−4.2	449.0944; 461.0851
2,3-Dehydrosilybin	C_25_H_20_O_10_	479.0941	10.0	−7.7	299.0178
Silychristin A	C_25_H_22_O_10_	481.1119	5.1	−3.3	125.0229; 325.0711
Silychristin B	C_25_H_22_O_10_	481.1115	5.3	−4.2	125.0253
Demethylation	C_24_H_18_O_10_	465.0816	5.9	−1.3	xxx
Reduction + demethylation	C_24_H_20_O_10_	467.0950	6.4	−6.0	xxx
Reduction + demethylation	C_24_H_20_O_10_	467.0934	6.1	−9.4	xxx
Reduction + demethylation	C_24_H_20_O_10_	467.0948	6.2	−6.4	xxx
2x reduction	C_25_H_24_O_10_	483.1307	5.3	3.3	125.0245
**2,3-Dehydrosilydianin**					
2,3-Dehydrosilydianin	C_25_H_20_O_10_	479.0955	7.0	−4.8	299.0168; 127.0477
Silydianin	C_25_H_22_O_10_	481.1135	6.3	−0.2	151.0013; 178.9931
Cysteine conjugation	C_28_H_25_NO_11_S	582.1053	8.2	−3	324.0479
Cysteine conjugation	C_28_H_25_NO_11_S	582.1066	9.1	−0.7	324.0489
Decarbonylation	C_24_H_20_O_9_	451.1004	7.1	−5.5	315.1245; 300.1013
Decarbonylation	C_24_H_20_O_9_	451.1029	9.2	0.0	301.0341
Demethylation	C_24_H_18_O_10_	465.0805	7.1	−3.7	xxx
Reduction + demethylation	C_24_H_20_O_10_	467.0966	6.2	−2.6	125.0244; 151.0105
Reduction + cysteine conjg.	C_28_H_27_NO_11_S	584.1221	7.7	−1	326.0655; 125.0227

MS^E^ refers to mass spectrometry experiment with higher collision energy without isolation on the quadrupole (see Section 3.6). xxx: MSE data were not measurable.
